# The complete chloroplast genome of *Striga asiatica* (L.) Kuntze 1891 (*Orobanchaceae*), a hemiparasitic weed from Guangxi China

**DOI:** 10.1080/23802359.2023.2197089

**Published:** 2023-04-10

**Authors:** Liu Qin, Enke Lu, Kexin Chen, Rizhen Bao, Lina Liang, Xiaohu Hu

**Affiliations:** a Key Laboratory for Conservation and Utilization of subtropical Bio-Resources Education Department of Guangxi Zhuang Autonomous Region, Yulin Normal University, Yulin, China; b Guangxi Key Laboratory of Agricultural Resources Chemistry and Biotechnology, Yulin Normal University, Yulin, China

**Keywords:** Chloroplast genome, Hemiparasitic plant, Orobanchaceae, *Striga asiatica*

## Abstract

*Striga asiatica* (L.) Kuntze 1891 is a hemiparasitic plant native to Asia and Africa. It is invasive and causes yield losses in crops such as corn, rice and sorghum. Lack of chloroplast genomic data has limited research into its obligate parasitic lifestyle. In this study, the complete chloroplast genome of *Striga asiatica* was sequenced and characterized. It is a quadripartite structure with a total length of 191,085 bp and a GC content of 37.86%. It has a large single copy region (LSC) of 51,406 bp, a small single copy region (SSC) of 273 bp, and two copies of the reverse repeat sequence (IRA and IRB) of 69,703 bp. A total of 122 protein-coding genes, 8 rRNA genes, and 44 tRNA genes were annotated in the chloroplast genome. There were a lot of *ndh* gene deletions and pseudogenizations in this chloroplast genome. For example, *ndh*A, D, E, H, I, and K were all pseudogenes because they were missing the 5′ end start codon. *ndh*B, C, and J had shorter gene lengths than their homologs, and *ndh*F and *ndh*G were missing genes. The phylogenetic tree reveals that all *Striga* species form a clade, and a bootstrap value of 100 indicates that *S. asiatica* is closely related to *Striga hermonthica* and *Striga sepera*. The comprehensive chloroplast genomic resource of *S. asiatica* would assist researchers in comprehending hemiparasitic mechanisms, molecular markers, and evolutionary patterns of the genus *Striga*.

## Introduction

*Striga asiatica* (L.) Kuntze 1891, a member of the Orobanchaceae family, is a hemiparasitic plant native to Asia and Sub-Saharan Africa (Cochrane and Press 1997). It is recognized as an invasive plant in many countries because its parasitism damages several major crops, including corn, rice, and sorghum, resulting in considerable yield losses (Spallek et al. 2013). In China and India, *S. asiatica* is used as folk medicine to treat high blood pressure, poor appetite, jaundice, and fever (Kakpure and Rothe 2012). Although the nuclear genome of *S. asiatica* has been sequenced (Yoshida et al. 2019), the lack of chloroplast genomic material has hampered research into *Striga asiatica*, particularly in terms of its obligate parasitic lifestyle. In this study, we obtained the complete chloroplast genome of *S. asiatica* using next-generation sequencing technology, characterized its basic features, and analyzed its phylogenetic relationship with other plants in the family Orobanchaceae.

## Materials and methods

**Figure 1.**
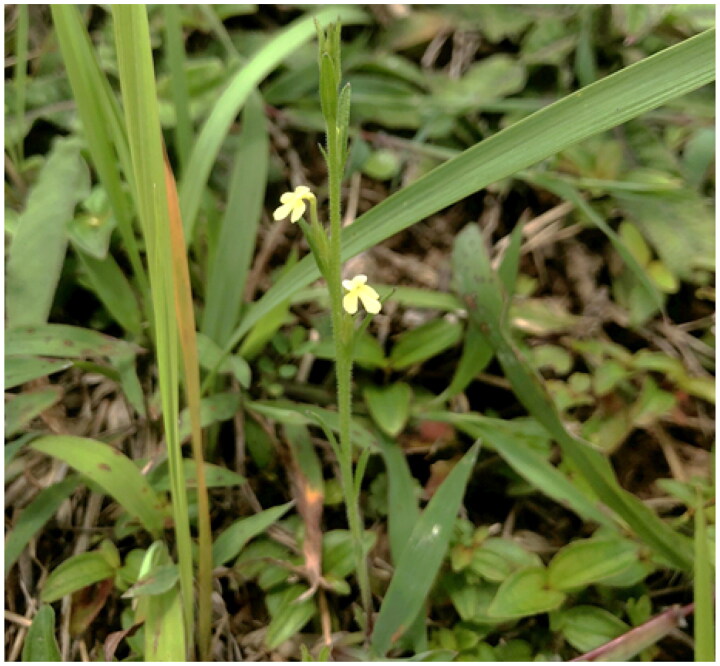
The morphological features of *Striga asiatica. S. asiatica* is an annual and semi-parasitic species, typified by its slender, striated leaves, spikes or individual blooms of yellow (occasionally tinged with red or white) with a deeply arched corolla tube, and an oval seed capsule that endures within a calyx bearing ten ridges. The photo was captured by RiZhen Bao in May of 2022 at Yulin Normal College, with no copyright concerns.

**Figure 2.**
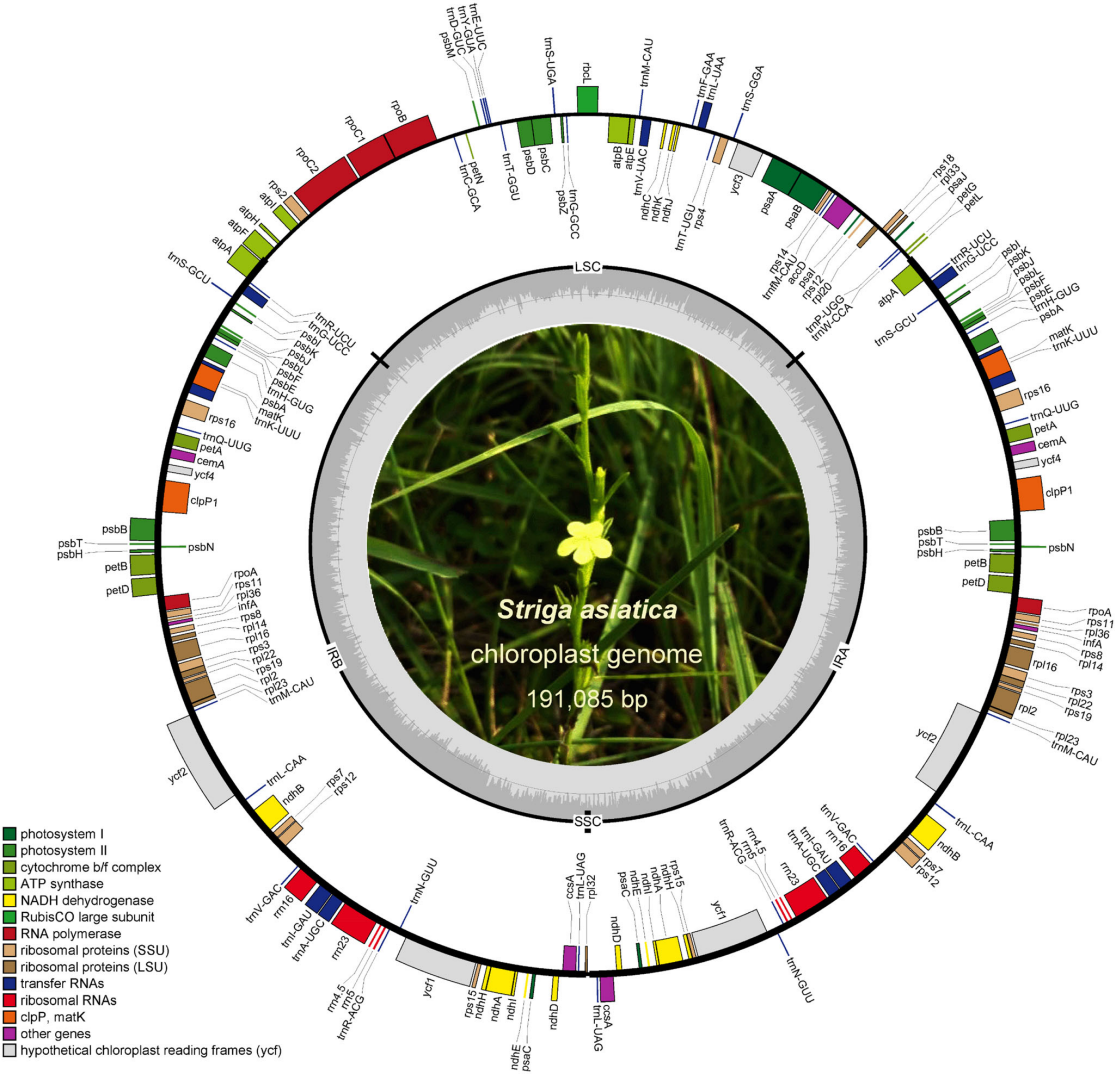
Chloroplast genome map of *Striga asiatica*. Different colors are used to highlight distinct gene functional groups. Indication for SSC, LSC and IR region are present. The center is a field photo of *S. asiatica.*

**Figure 3.**
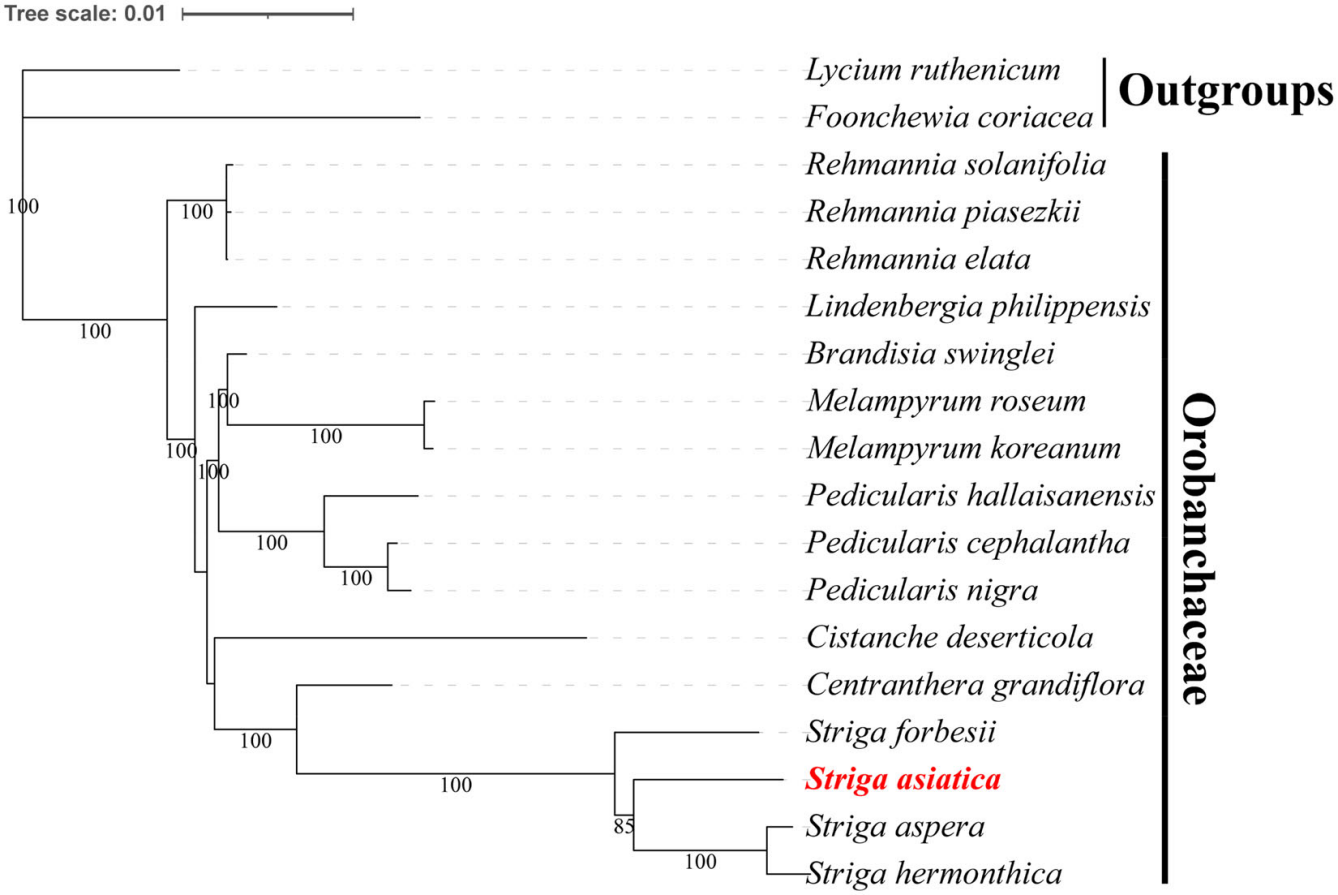
The Maximum likelihood (ML) phylogenetic tree is based on chloroplast genome of *S. aisatica* and other 17 species. The best-fit model is chosen as TVM + F + I + G4. Bootstrap values are based on 1000 replicates. The numbers on branches are bootstrap support values. *Striga asiatica* is highlighted in red and bold. The following sequences were used: *Striga hermonthica* MF780874.1 (Frailey et al. 2018), *Striga forbesii* MF780873.1 (Frailey et al. 2018), *Striga aspera* MF780872.1 (Frailey et al. 2018), *Striga asiatica* ON652844.1 (this study), *Rehmannia elata* KX636161.1 (Zeng et al. 2017), *Rehmannia piasezkii* KX636160.1 (Zeng et al. 2017), *Rehmannia solanifolia* KX636159.1 (Zeng et al. 2017), *Pedicularis cephalantha* OL606628.1 (Wang et al. 2022), *Pedicularis nigra* OL544940 (Wang et al. 2022), *Pedicularis hallaisanensis* MG770330 (Cho et al. 2018), *Melampyrum koreanum* MW463054 (Jin et al. 2021), *Melampyrum roseum* MN075942 (Yong-Chao et al. 2019), *Lindenbergia philippensis* HG530133.1 (Wicke et al. 2013), *Brandisia swinglei* MK381315.1 (Xia et al. 2019), *Centranthera grandiflora* MW262988 (Zheng and Li 2021), *Cistanche deserticola* KC128846.1 (Li et al. 2013), *Lycium ruthenicum* MK994503.1 (Wang et al. 2019), *Foonchewia coriacea* MT942688 (Zhang et al. 2021).

The fresh leaves of *S. asiatica* (Figure 1) were collected from the Yulin Normal University Horticultural Germplasm Center located in Yulin, Guangxi, China (110.185°E, 22.669°N). The voucher specimen (DJJ-YNU-001) was deposited in the Herbarium of Yulin Normal University (https://syy.ylu.cn/index.html, Yulin Zhu, gxzyl@163.com). The total genomic DNA was isolated using the DNeasy Plant Mini Kit (Qiagen, Hilden, Germany). Biozeron (Biozeron, Shanghai, China) was entrusted with the library preparation and sequencing utilizing the Illumina Hiseq 4000 sequencing technology (Illumina, San Diego, CA). The adapter removal and quality control of raw data was conducted by Trimmomatic v0.39 by using the following parameters: ‘ILLUMINACLIP: TruSeq3-PE-2.fa:2:30:10:8:true LEADING:4 TRAILING:4 SLIDINGWINDOW:4:110 MINLEN:100.’ The *de novo* assembling of the complete chloroplast genome was achieved by using the GetOrganelle toolkit with default parameters (Jin et al. 2020). To assess the quality of the resulting chloroplast genome assembly, we applied the ‘evaluate_assembly_using_mapping.py’ script from the aforementioned toolkit, which allowed us to accurately calculate the overall coverage depth (Figure S1). With the help of CPGAVAS2 (Shi et al. 2019) and GeSeq (Tillich et al. 2017), the chloroplast genome of the hemiparasitic *Pedicularis hallaisanensis* (Orobanchaceae) was used as a guide to annotate the chloroplast genome of *Striga asiatica* in detail. The final sequence and annotation file was summitted to NCBI with the accession of ON652844.1. The OGDRAW (Greiner et al. 2019) was employed to convert the chloroplast genome annotation into graphical map (Figure 2). To visualize the structures of splicing genes within the chloroplast genome of *S. asiatica* (Figure S2 and S3), we utilized the CPGView tool (Liu et al. 2023). The study utilized a total of 16 complete chloroplast genome sequences from the Orobanchaceae family and 2 from other families in order to reconstruct a phylogenetic tree and establish the taxonomic status of *S. asiatica*. MAFFT v7 (Rozewicki et al. 2019) was used to align the single-copy orthologue genes of all chloroplast genomes with default parameters, and the ModelFinder in IQ-TREE v1.6.10 (Nguyen et al. 2015) was used to perform model detection. With the best-fit model TVM + F + I + G4 and two outgroup species from the *Solanaceae*, *Agastache rugosa* and *Callicarpa americana*, the maximum likelihood (ML) tree with 1000 bootstraps was constructed (Figure 3).

## Results and discussion

The complete chloroplast genome of *S. asiatica* was obtained, with a length of 191,085 bp and a GC content of 37.86%. The genome is composed of a large single-copy region (LSC) of 51,406 bp, a small single-copy region (SSC) of 273 bp, as well as two copies of the inverted-repeat regions (IRa and IRb) of 69,703 bp. The IR region expansion, which resulted in the enlargement of the chloroplast genome, occurred in both the LSC and SSC regions. This type of IRs increases, also observed in other *Striga* chloroplast genomes, may be unique to particular lineages (Frailey et al. 2018). A total of 174 genes were annotated, comprising 122 protein-coding genes (PCGs), 8 ribosomal RNA (rRNA) genes, and 44 transfer RNA (tRNA) genes. As a result of the expansion of IR regions, 46 genes have duplicated copies. Numerous *ndh* genes were detected as pseudogenized, including *ndh*A, D, E, H, I, and K, which lacked a 5′-terminal start codon. Moreover, despite possessing complete open reading frames, *ndh*B, C, and J had significantly shorter gene lengths than their respective homologs. In addition, *ndh*F and *ndh*G, were identified as missing genes. The phenomenon of *ndh* gene loss and pseudogenization had been observed extensively in other parasitic plants (Xu et al. 2020; Li et al. 2021).

The phylogenetic tree reveals that all *Striga* species constitute a clade, and *S. asiatica* is closely related to *Striga hermonthica* and *Striga sepera* with the bootstrap value of 100 (Figure 3). The congruity observed between the phylogenetic outcomes of this investigation and those derived by Frailey et al. (2018) and Zeng et al. (2017) reinforces the current understanding of the evolutionary relationships among *Striga* species, and thereby advances our comprehension of the genetic diversity and evolutionary history of these parasitic plants.

## Conclusion

In conclusion, the sequencing and analysis of the complete chloroplast genome of *Striga asiatica* using next-generation sequencing technology has provided valuable information about its genetic features and evolutionary relationships with other plants in the Orobanchaceae family, which can contribute to a better understanding of the parasitic mechanisms, molecular markers, and evolutionary patterns of the *Striga* genus.

## Supplementary Material

Supplemental Material

## Data Availability

The genome sequence data that support the findings of this study are openly available in GenBank of NCBI at https://www.ncbi.nlm.nih.gov under the accession no. ON652844.1. The associated BioProject, SRA, and Bio-Sample numbers are PRJNA892165, SRR22000393, and SAMN31370639, respectively.
