# Supplementary material for: The complete chloroplast genome of Striga asiatica (L.) Kuntze 1891 (Orobanchaceae), a hemiparasitic weed from Guangxi China
Source: Mitochondrial DNA B Resour. 2023 Apr 10;8(4):497–500. doi: 10.1080/23802359.2023.2197089 (PMC10101682; doi:10.1080/23802359.2023.2197089)
Supplement: Supplemental Material [file TMDN_A_2197089_SM8378.docx]

Figure captions

Figure S1. Overall coverage depth of the chloroplast genome assembly of *Striga asiatica*.

Figure S2. Schematic map of the cis-splicing genes in the chloroplast genome of *Striga asiatica*.

Figure S3. Schematic map of the trans-splicing genes in the chloroplast genome of *Striga asiatica*


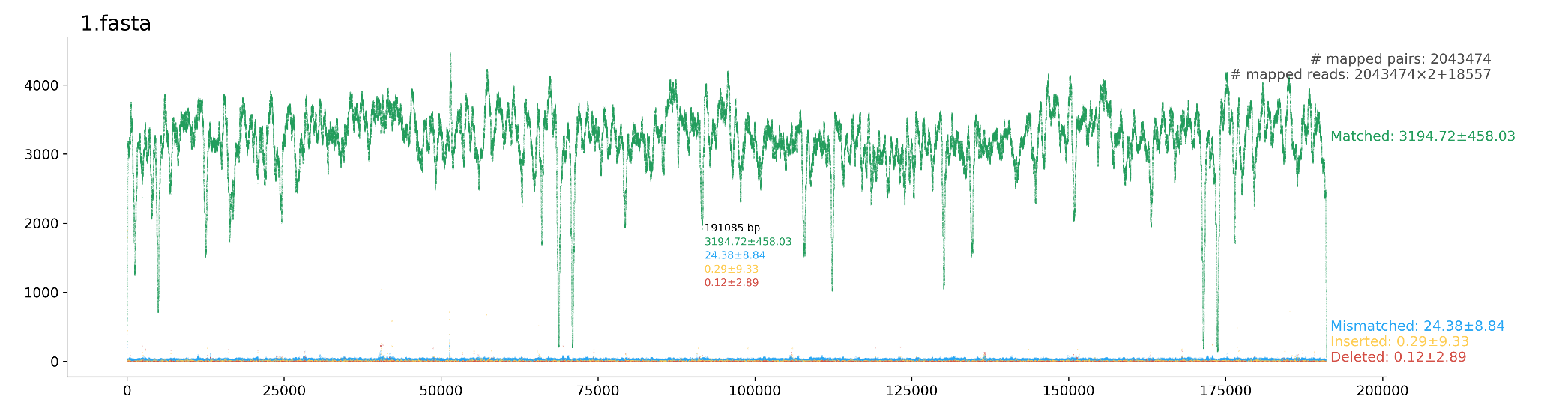


Figure S1. Overall coverage depth of the chloroplast genome assembly of *Striga asiatica*. The script “evaluate_assembly_using_mapping.py” from the GetOrganelle toolkits calculated the average and standard deviation of matched depth at each site/site-interval across the whole assembly and generated the plot using Python library Matplotlib.


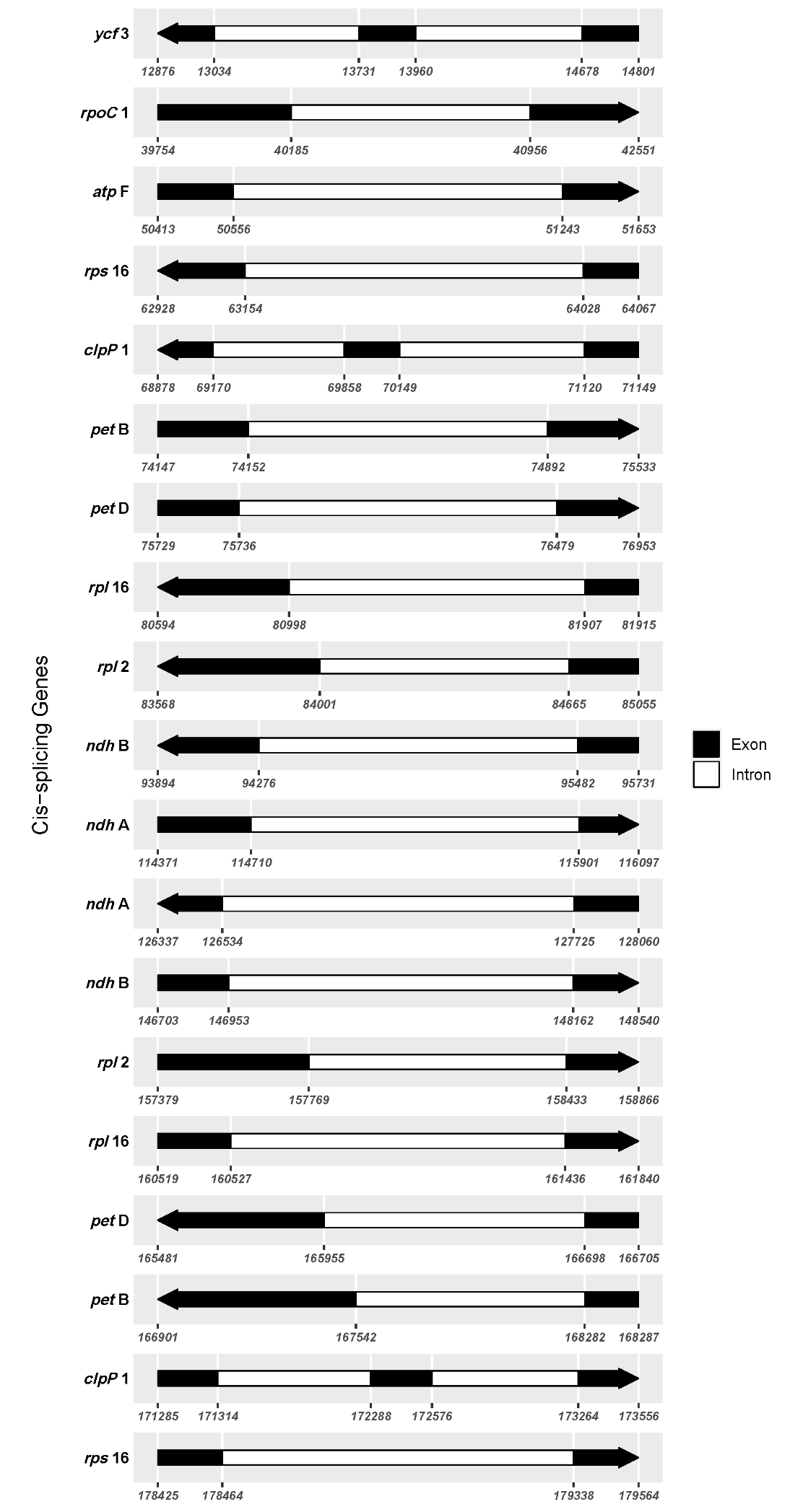


Figure S2. Schematic map of the cis-splicing genes in the chloroplast genome of *Striga asiatica*. This figure illustrates the schematic map of cis-splicing genes found in the chloroplast genome. The genes are organized in a top-to-bottom manner based on their sequence on the chloroplast genome, and are labeled on the left side, while their structures are depicted on the right side. Exons are represented in black, and introns in white. The direction of the gene is indicated by an arrow.


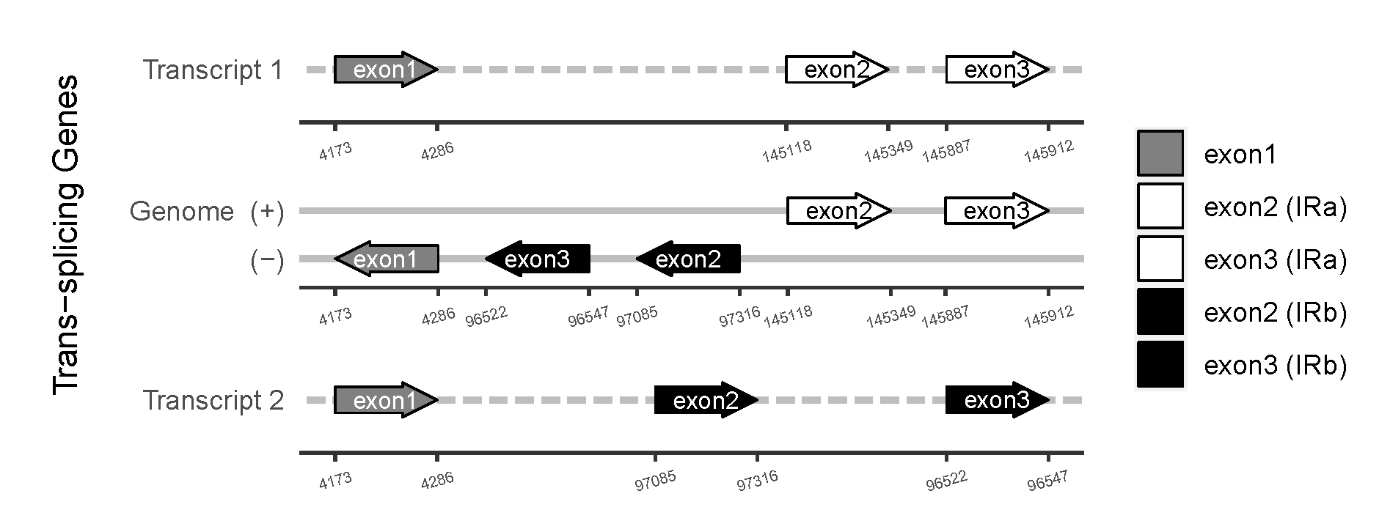


Figure S3. Schematic map of the trans-splicing genes in the chloroplast genome of *Striga asiatica*. This figure displays the schematic map of the trans-splicing genes present in the chloroplast genome of *Striga asiatica*, with a focus on the trans-splicing gene *rps*12. The gene comprises three distinct exons, with two of them being duplicated as they are located in the inverted repeat (IR) regions.
